# Helicobacter pylori infection related long noncoding RNA (lncRNA) AF147447 inhibits gastric cancer proliferation and invasion by targeting MUC2 and up-regulating miR-34c

**DOI:** 10.18632/oncotarget.13165

**Published:** 2016-11-07

**Authors:** Xiaoying Zhou, Han Chen, Li Zhu, Bo Hao, Weifeng Zhang, Jie Hua, Huiyuan Gu, Wujuan Jin, Guoxin Zhang

**Affiliations:** ^1^ Department of Gastroenterology, First Affiliated Hospital of Nanjing Medical University, Nanjing, China; ^2^ First Clinical Medical College of Nanjing Medical University, Nanjing, China; ^3^ Department of Gastroenterology, People's Hospital of Jingjiang, Taizhou, China; ^4^ Department of Gastroenterology, First Affiliated Hospital of Soochow University, Suzhou, China

**Keywords:** helicobacter pylori infection, lncRNA, gastric cancer, MUC2

## Abstract

Long non-coding RNAs (lncRNAs) were shown to play critical roles in cancer biology. We investigated whether *H. pylori* infection could promote gastric cancer by regulating lncRNAs expression. Differentially expressed lncRNAs between *H. pylori* positive and negative tissues were identified by microarray and validated by qRT-PCR. Our results indicated that *H. pylori* positive tissues have a specific profile of lncRNAs. Cell biological assays with siRNA-mediated knockdown or lentivirus vector-mediated over-expression were performed to probe the functional relevance of the lncRNAs. We identified an lncRNA-AF147447 decreased expressed by *H. pylori* infection, which can inhibit GC proliferation and invasion *in vitro* and *in vivo*, act as a tumor suppressor in the development of *H. pylori* induced GC. LncRNA AF147447 could repress MUC2 expression by direct binding or increasing miR-34c expression. We also found that transcription factor E2F1 could be recruited to lncRNA AF147447 promoter by RNA immunoprecipatation and RNA pull down assays. These findings support a role of lncRNA AF147447 in tumor suppression. This discovery contributes to a better understanding of the importance of the deregulated lncRNAs by *H. pylori* infection and provides a rationale for the potential development of lncRNA-based targeted approaches for the treatment of *H. pylori*-related gastric cancer.

## INTRODUCTION

Gastric cancer (GC) belongs to the third leading cause of cancer-related death in the worldwide [1]. A lot of risk factors contribute to GC, among which Helicobacter pylori (*H. pylori*) is the strongest one [2]. The rate of *H. pylori* infection is over 50% worldwide nowadays, especially among developing countries [3]. The mechanism considering how *H. pylori* infection induces GC has not been fully identified [4].

Long non-coding RNAs (LncRNAs) are a group of non-coding RNA transcripts which are longer than 200 nucleotides (nt) [5]. Normally, they have little or no protein capacity. Like miRNAs, lncRNAs play important roles in regulating target gene expression, by transcription, posttranscriptional processing or epigenetically regulation, including binding with miRNA or chromatin modification and genomic imprinting [6], and therefore, lncRNAs are associated with various diseases, such as metabolic disorders [7], cardiac diseases [8], tumors [9], etc.

Numerous studies have suggested the role of dysregulated lncRNA expression in GC. Zhang et al [10] found that lncRNA ANRIL was significantly upregulated in GC tissues. By recruiting and binding to PRC2, ANRIL epigenetically repress miR-99a/miR-449a, like ceRNAs, in controlling the targets-mTOR and CDK6/E2F1 pathway. Xu et al [11] found that lncRNA FENDRR expression was down-regulated in both GC cell lines and tissues and it could be considered as diagnostic and prognostic markers for GC patients. However, until now, no studies were performed regarding the dysregulated lncRNAs in *H. pylori* infection-related GC.

In this study, we were the first to explore the dysregulated lncRNA expression in *H. pylori* positive tissues by lncRNA microarray. Our study showed that numbers of lncRNAs were dysregulated. We further investigated the detailed function of a *H. pylori* infection-related down-regulated lncRNA, AF147447, *in vivo* and *in vitro*, and found that it plays an important role in *H. pylori*-related GC.

## RESULTS

### Differential LncRNA Expressions were found in the gastric tissues of *H. pylori* positive and negative patients

We chose 3 matching pairs of *H. pylori* infected and non-infected gastric tissues for microarray analysis of lncRNAs and mRNAs. We set a threshold as a fold change that is larger than 2.0 and a *p value* that is smaller than 0.05 and found that there were 123 dysregulated lncRNAs and 87 dysregulated mRNAs in the gastric tissues of *H. pylori* infected and non-infected patients, which means that the lncRNA (Supplementary Figure S1A) and mRNA (Supplementary Figure S1B) expression level were significantly different between the two groups. In order to validate microarray analysis findings, top ten lncRNAs from the differentially expressed lncRNAs were randomly selected with a fold change that is larger than 5 and detected their expressions by real time (RT)-PCR. The PCR validation data were the same as the microarray results (Supplementary Figure S1C, *p* < 0.05 for all). Consequently, our results suggested that a set of lncRNAs were frequently dysregulated in the gastric tissues of *H. pylori* infected and control patients and they may be related to the *H. pylori*-related carcinogenesis.

### LncRNA AF147447 was decreased expressed in *H. pylori* infected tissues and cells

**Figure 1:**
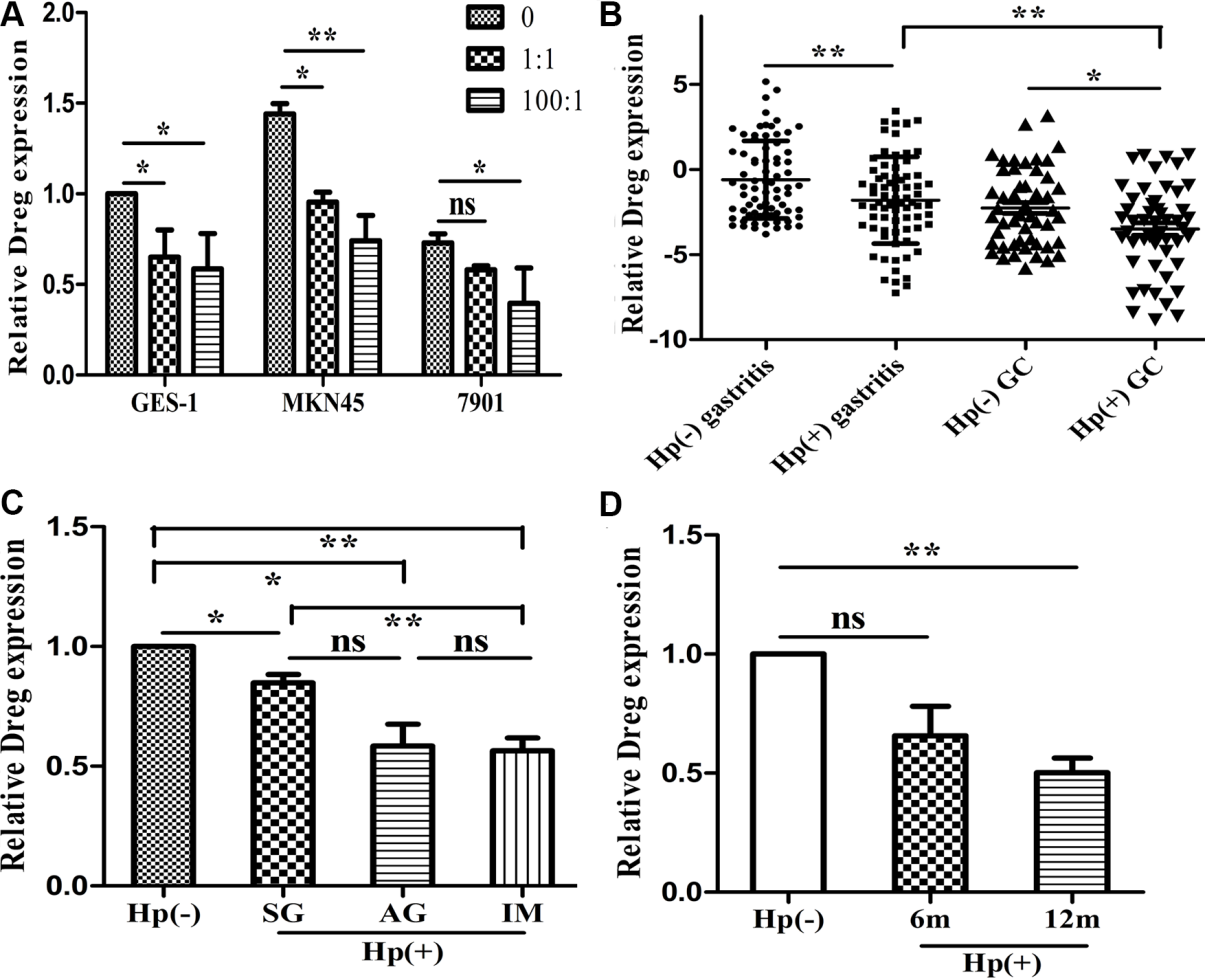
LncRNA AF147447 expression in *H. pylori* infected state (**A**) AF147447 expression in *H. pylori* infected cells compared with controls. (**B**) AF147447 expression in *H. pylori* infected gastritis and GC tissues compared with their respective controls. (**C**) AF147447 expression in different histology type of *H. pylori* infected gastritis. (**D**) AF147447 expression in *H. pylori* infected gastric tissues in mice. (*p < 0.05, **p < 0.01).

Among the lncRNAs differentially expressed in tissues, we found that an lncRNA, accession number AF147447 in NCBI, was significantly decreased by *H. pylori* infection. Its expression decreased most significantly in qRT-PCR analysis (Supplementary Figure S1C). Consequently, we chose this lncRNA for further functional analysis and investigate potential mechanisms underlying *H. pylori* induced GC. In order to analyze *in vitro* whether *H. pylori* infection alters AF147447 expression, we measured AF147447 expression after *H. pylori* co-culture with GC cells. We found that AF147447 expression was decreased dose-dependently with *H. pylori* infection MOIs in three gastric epithelial cells (Figure 1A). Then, we detected AF147447 expression in 75 pairs of *H. pylori* infected and non-infected tissues and 50 pairs of tumor tissues by qRT-PCR. The basic characteristics of enrolled patients were shown (Supplementary Table S1) and the results showed that AF147447 was significantly decreased when *H. pylori* infected, regardless of control or tumor tissues. Additionally, we also found out that the expression of AF147447 was significantly decreased in tumor tissues (Figure 1B). When compared its expression with clinical characteristics, we found that patients with intestinal metaplasia expressed significantly lower AF147447 compared with superficial gastritis or atrophic gastritis (Figure 1C), which suggested that the more severe the histology of the patients is, the lower AF147447 expressed. In 50 pairs of tumor tissues, we found that patients with advanced stages showed significantly lower AF147447 expression (Supplementary Figure S2). We next examined AF147447 expression in *H. pylori* infected gastric tissues in mice. Infected mice lasted for one year yielded the lowest AF147447 expression (Figure 1F), which showed that AF147447 expression was decreased both time dependently and dose dependently with *H. pylori* infection.

### Cell proliferation and cell migration were inhibited by lncRNA AF147447 *in vitro* **and** *in vivo*

**Figure 2:**
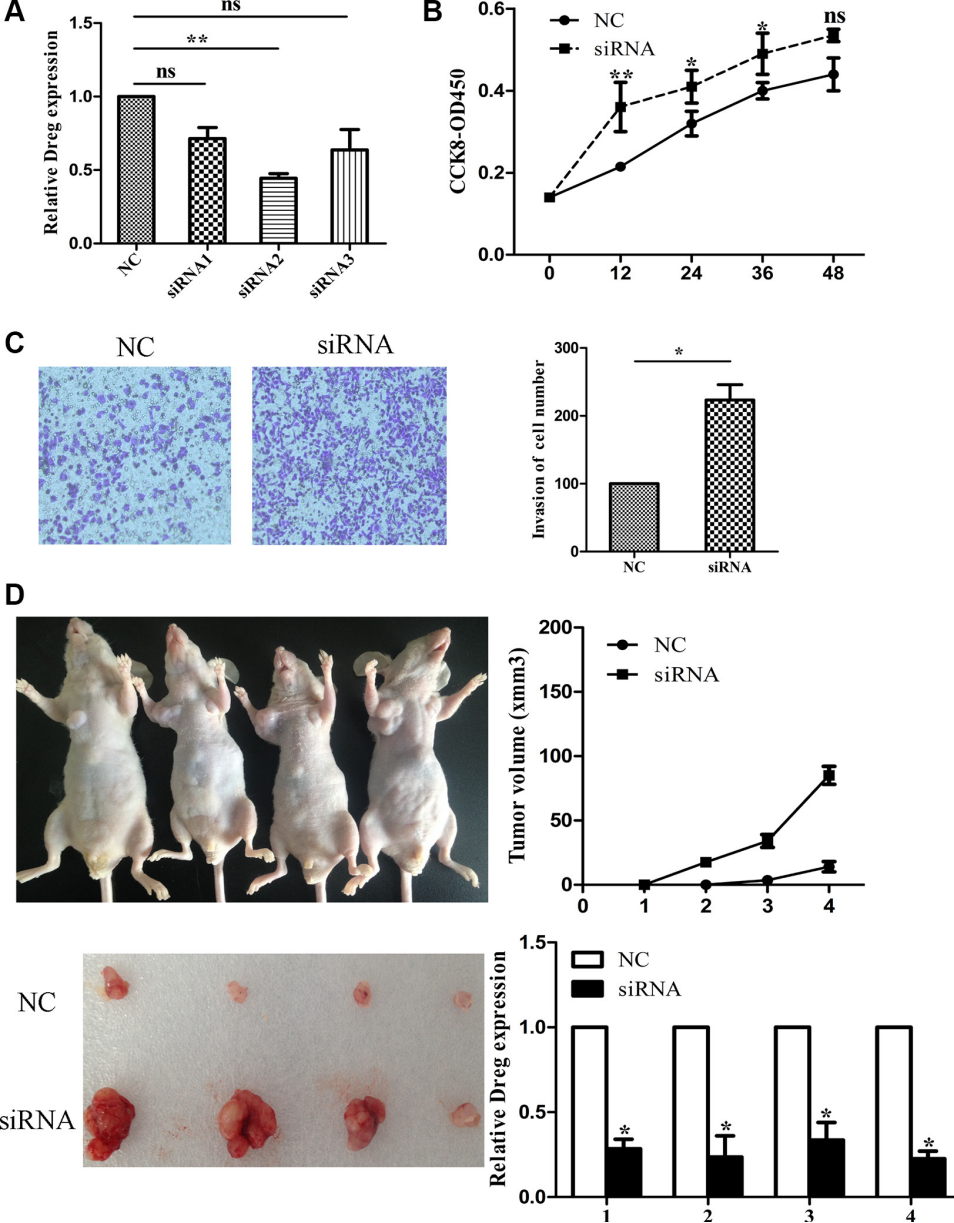
LncRNA AF147447 inhibition promote GC proliferation and invasion *in vitro* **and** *in vivo* (**A**) LncRNA Af147447 expression level after AF147447 siRNA transfection was validated by qRT-PCR. (**B**) MKN45 cells were seeded in 96-well plates, and cell proliferation was assessed daily for 3 days using the Cell Counting Kit-8 (CCK-8) assay. (**C**) Cells were treated with either AF147447 siRNA or siRNA-NC for 24 h. The representative images of invasive cells at the bottom of the membrane stained with crystal violet were visualized as shown. All experiments were performed in triplicate. (**D**) The *in vivo* models used were xenograft-transplanted nude mouse tumor models of human gastric cancer established with Af147447 siRNA or siRNA control. The left arms of the mice were injected with control GC cell and the right arms of the mice were injected with AF147447 siRNA GC cells. Photographs of tumors are presented. Effects of lncRNA AF147447 expression in nude mouse models are shown. (*p < 0.05, **p < 0.01).

LncRNA AF147447 down-regulation in *H. pylori* positive tissues suggested that it may play an important role in *H. pylori* linked carcinogenesis process. Thus, we investigated cell proliferation, migration, and invasion by decreasing or increasing AF147447 expression. Firstly, we examined the expression of AF147447 in 5 gastric epithelial cells. We found that AF147447 expression was lowest in 7901 and highest in MKN45 (Supplementary Figure S1C). Consequently, we chose 7901 to over-express AF147447 and MKN45 to reduce AF147447 expression. 3 siRNAs were used to interfere with AF147447 expression and siRNA2 showed the highest inhibition rate (Figure 2A). We chose siRNA2 for further functional analysis. From CCK8 analysis, we found that suppression of AF147447 greatly enhanced cell proliferation (Figure 2B). Also, AF147447 inhibition also promoted GC cells invasion activity compared with controls (Figure 2C). We also found *in vivo* that comparing to those injected with siRNA controls, mice injected with AF147447 siRNA exhibited significantly larger tumor volume and faster tumor growth (Figure 2D). We then extracted RNA from the tumors and found that AF147447 expression was significantly lower after siRNA injection by qRT-PCR analysis.

**Figure 3:**
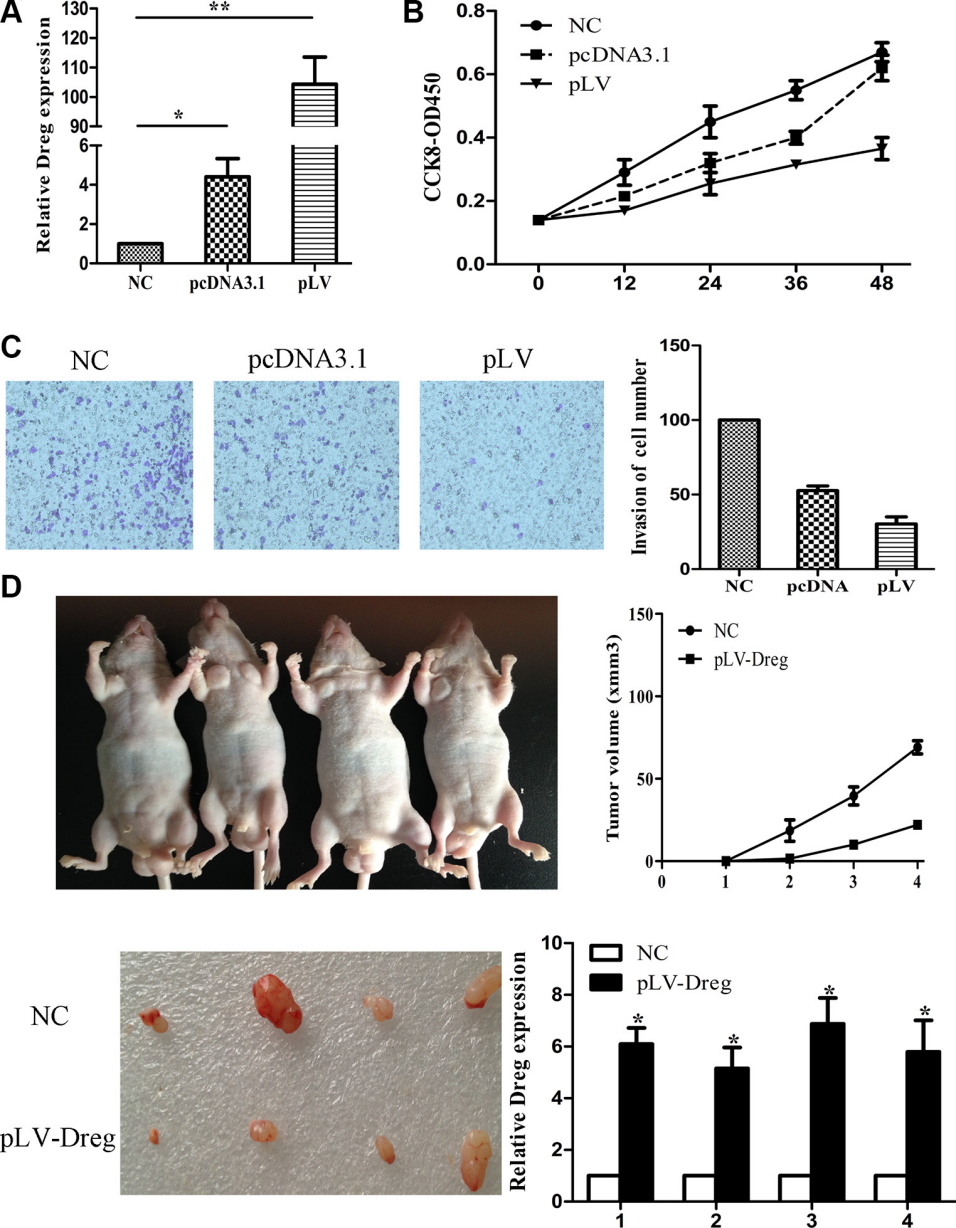
LncRNA AF147447 over-expression inhibit GC proliferation and invasion *in vitro* **and** *in vivo* (**A**) Af147447 expression level after AF147447 over-expression was validated by qRT-PCR. (**B**) 7901 cells were seeded in 96-well plates, and cell proliferation was assessed daily for 3 days using the Cell Counting Kit-8 (CCK-8) assay. (**C**) Cells were treated with either pcDNA-AF147447 or pLV-AF147447 for 24 h. The representative images of invasive cells at the bottom of the membrane stained with crystal violet were visualized as shown. All experiments were performed in triplicate. (**D**) The *in vivo* models used were xenograft-transplanted nude mouse tumor models of human gastric cancer established with pLV-AF147447 or pLV-NC. The left arms of the mice were injected with pLV-AF147447 GC cells and the right arms of the mice were injected with pLV-NC cells. Photographs of tumors are presented. Effects of lncRNA AF147447 expression in nude mouse models are shown. (*p < 0.05, **p < 0.01).

Next, we over-expressed AF147447 by pcDNA or lentivirus. We found that after pcDNA transfection or lentivirus infection, the expression of AF147447 was significantly up-regulated (Figure 3A). From CCK8 analysis, we found that over-expression of AF147447 significantly inhibit cell proliferation (Figure 3B). It also inhibited GC cells invasion activity after pcDNA transfection or lentivirus infection compared with controls (Figure 3C). For nude mice xenoplantation, GC cells transfected with pLV-AF147447 or control were injected. Mice which were injected with pLV-AF147447 exhibited much lower tumor growth and less tumor volume compared with mice injected with controls (Figure 3D). AF147447 expression from the tumors was significantly higher in pLV- AF147447 transfected mice by qRT-PCR analysis. Together, these results showed that, both *in vitro* and *in vivo*, lncRNA AF147447 inhibited cell proliferation and migration.

### LncRNA AF147447 could bind with and target oncogene MUC2

**Figure 4:**
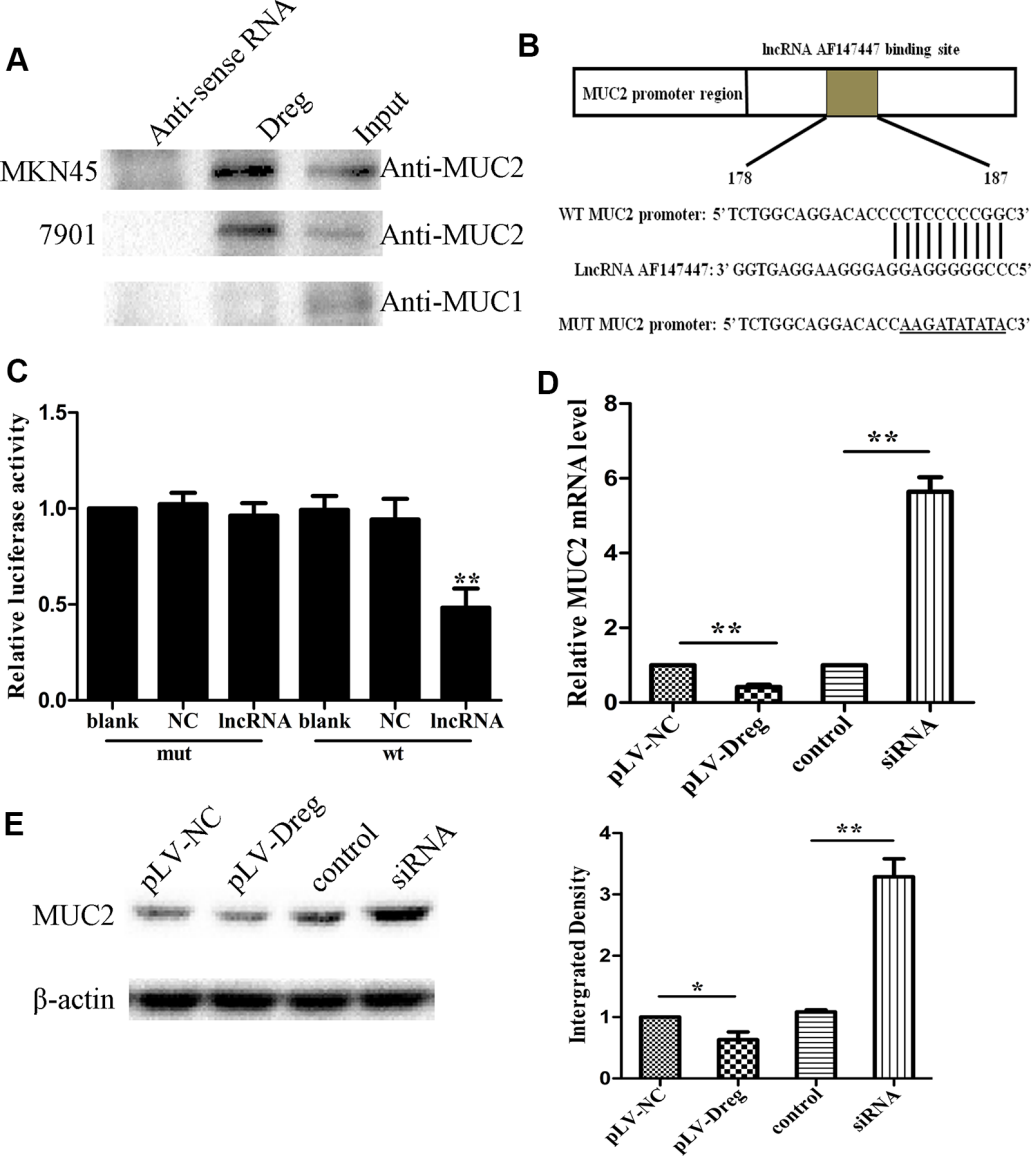
Identification and validation of lncRNA-AF147447 targets (**A**) Biotinylated lncRNA-AF147447 or antisense RNA were incubated with nuclear extracts, targeted with streptavidin beads, and washed, and associated proteins were resolved in a gel. Western blotting analysis of the specific association of MUC2 and lncRNA-AF147447. Another MUC family: MUC1 is shown as a control. (**B**) Diagram of MUC2 promoter region constructs. (**C**) Luciferase reporter assays in 7901 cells, with co-transfection of wt or mt and lncRNA as indicated. (**D**) MUC2 expression was validated by qRT-PCR after transfecting with pLV-AF147447 or siRNA or their respective controls. (**E**) MUC2 expression was validated by western blot after transfecting with pLV-AF147447 or siRNA or their respective controls. (*p < 0.05; **p < 0.01).

Studies have been suggested that lncRNAs play their regulatory roles in inducing tumorigenesis mainly by binding with their target gene products. Consequently, in order to figure out whether this lncRNA could function by this way, RNA pull-down analysis was therefore performed to identify all the possible proteins that were bind with AF147447 (Figure 4A). MUC2 was therefore, revealed by this mass spectrometry analysis, specifically associated with AF147447 among one of the many binding proteins (Supplementary Table S2).

Previous studies showed that MUC2 overexpression was related to GC progression and poor prognosis. In order to validate the relationship between AF147447 and MUC2, luciferase reporter assay was performed with MUC2 promoter region. LncRNA AF147447 could combine with MUC2 promoter region through sequence match (Figure 4B). The target sequence of the mutant (mt) or wild type (wt) promoter was cloned into a luciferase reporter vector. We then transfected the cells with wt/mt vector together with lncRNA AF147447. We found that cells transfected with wt promoter presented with a marked decrease of luciferase activity, comparing with those transfected with NC (Figure 4B, *p* < 0.01). However, the luciferase activity of mt vector remained the same after a simultaneous transfection with AF147447.

Then, the functional relevance between lncRNA AF147447 and MUC2 was determined. LncRNA AF147447 could inhibit or promote cell proliferation and invasion through targeting and changing the expression of MUC2. In order to test this hypothesis, we measured the RNA (Figure 4C) and protein (Figure 4D) expression levels of MUC2 by qRT-PCR and western blot in 7901 cells with AF147447 overexpression or decreased expression. We found that MUC2 expression was markedly decreased in the cells stably transfected with pLV-AF147447 than that with pLV-NC, and was significantly increased transfecting with AF147447 siRNA compared with those transfecting with the siRNA control. These results suggested that AF147447 could inhibit MUC2 RNA and protein expression.

**Figure 5:**
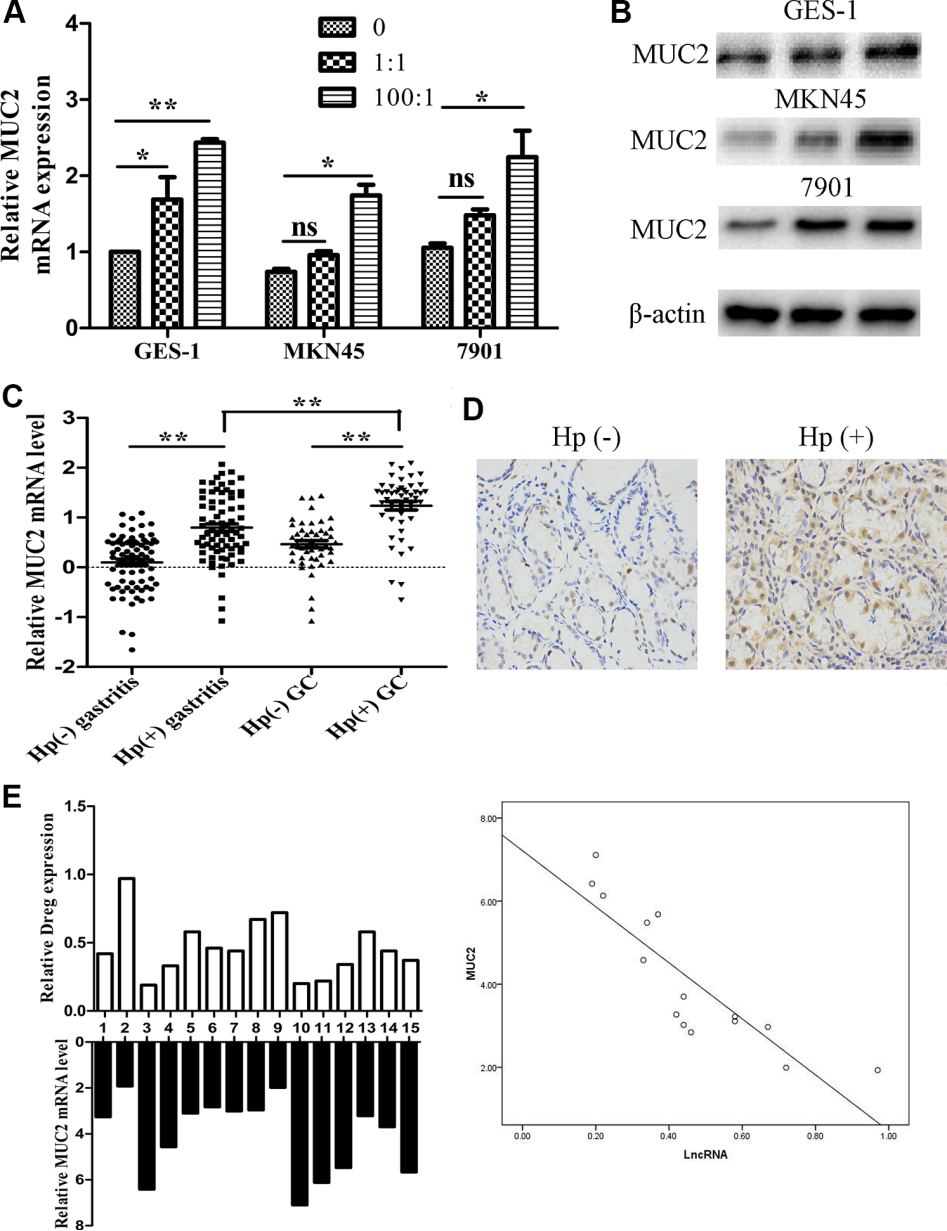
MUC2 expression under *H. pylori* infected state (**A**) MUC2 mRNA expression in *H. pylori* infected cells compared with controls. (**B**) MUC2 protein expression in *H. pylori* infected cells compared with controls. (**C**) MUC2 mRNA expression in *H. pylori* infected control and tumor tissues. (**D**) MUC2 protein expression in *H. Pylori* positive and negative tissues was detected by immunohistochemical staining. (**E**) A statistically significant inverse correlation between MUC2 and lncRNA-AF147447 levels in clinical specimens (Spearman's correlation analysis, r = −0.864, *p* = 0.000) (*p < 0.05; **p < 0.01).

We also determined MUC2 RNA and protein levels after cells infected with different density of *H. pylori* bacteria. After *H. pylori* infection, the infected cells showed markedly increased MUC2 RNA and protein expression (Figure 5A and 5B). Then, we detected its expression both in 75 pairs of *H. pylori* infected and non-infected tissues and 50 pairs of tumor tissues by qRT-PCR. The results showed that MUC2 expression was markedly higher in infected gastric tissues, especially in infected tumor tissues (Figure 5C). Immunohistochemistry analysis also suggested that MUC2 expression was up-regulated in the infected tissues (Figure 5C). We selected 15 tissues and detected AF147447 and MUC2 expression respectively. From pearson correlation analysis, we could conclude that MUC2 expression was negatively correlated with AF147447 (r = −0.864, *p* = 0.000) (Figure 5D).

### LncRNA AF147447 regulate miR-34c expression which could target MUC2

**Figure 6:**
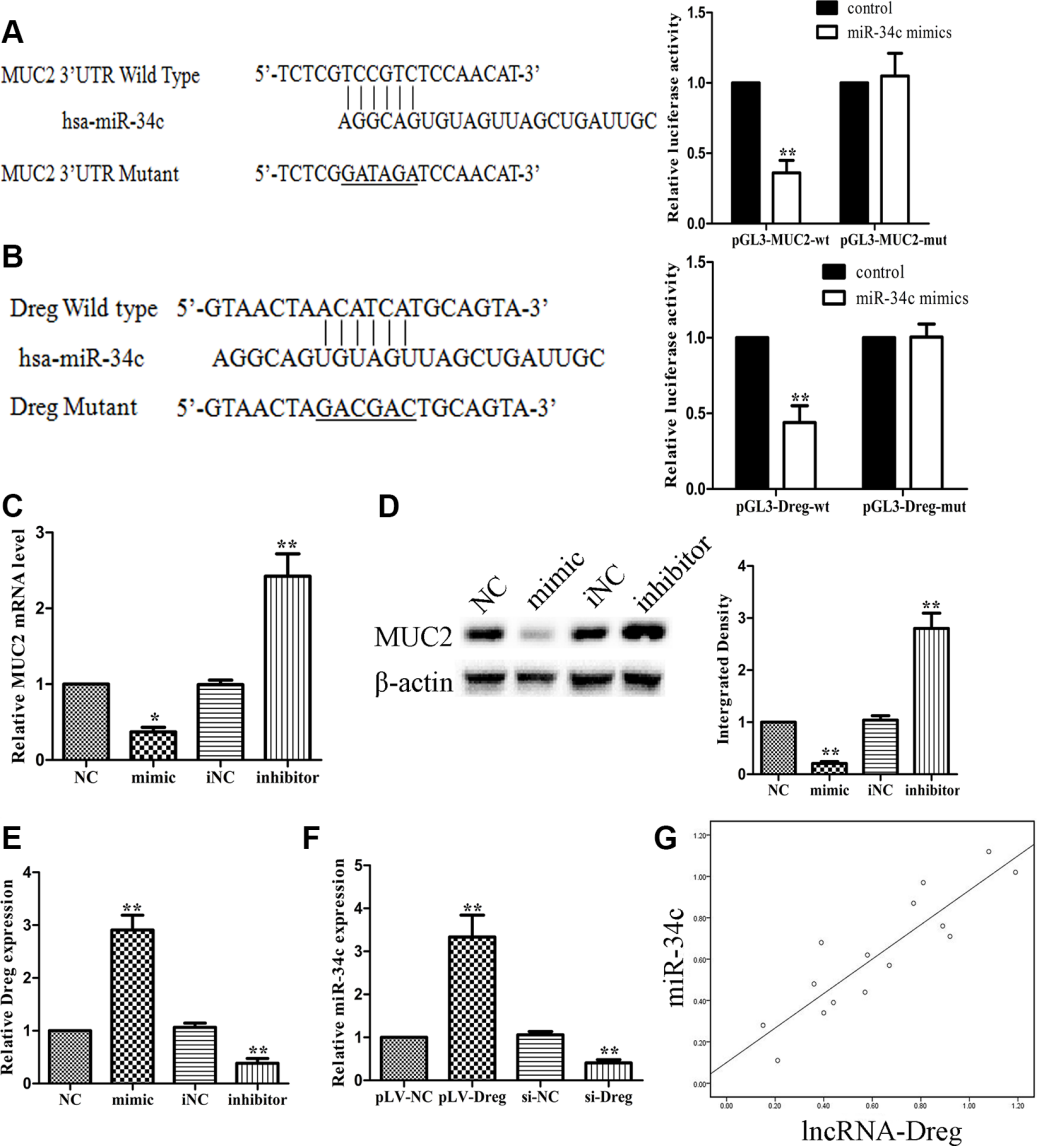
LncRNA-AF147447 interact with increased miR-34c expression level (**A**) Diagram of MUC2 3′UTR region constructs. Luciferase reporter assays in 7901 cells, with co-transfection of miR-34c mimics and wt or mut 3′UTR. (**B**) Diagram of AF147447 wt and mut constructs. Luciferase reporter assays in 7901 cells, with co-transfection of miR-34c mimic and AF147447 wt or mut. (**C**) MUC2 mRNA expression was validated by qRT-PCR after cells transfecting with mimic or inhibitor. (**D**) MUC2 protein expression was validated by western blot after cells transfecting with mimic or inhibitor. (**E**) AF147447 expression was validated by qRT-PCR after cells transfecting with mimic or inhibitor. (**F**) MiR-34c expression was validated by qRT-PCR after cells transfecting pLV-AF147447 or si-AF147447. (**G**) Pearson analysis showed that miR-34c and lncRNA AF147447 was positively correlated. (*p < 0.05; **p < 0.01).

We further investigated whether lncRNA AF147447 could alter miRNA expression and thus affect MUC2 expression. Firstly, we searched from miRanda database and found that miR-34c might be one regulatory gene of MUC2. We constructed luciferase gene and found that miR-34c mimic transfection significantly reduced the luciferase activity, which confirmed that MUC2 is miR-34c target gene (Figure 6A). By searching online databases, including PicTar, miRbase and BiBi Serv, the co-regulation of miRNA with lncRNA AF147447 was predicted by the bioinformatics analysis. The binding of miR-34c with lncRNA AF147447 was confirmed with the luciferase reporter assay (Figure 6B). We transfected with miR-34c mimics or inhibitor and found that MUC2 mRNA and protein (Figure 6C) were significantly decreased after transfecting with miR-34c mimics and increased after transfecting with inhibitor. We then co-transfected with mimic and si- AF147447 and found that the inhibition of MUC2 expression by miR-34c mimic was abrogated after si-AF147447 transfected. We also co-transfected with inhibitor and pLV-AF147447 and found that MUC2 overexpression was overcame after pLV-AF147447 transfected (Supplementary Figure S3A and S3B). We next examined the expression level of AF147447 after transfecting with miR-34c mimic or inhibitor. AF147447 was higher when miR-34c over-expressed and vice versa (Figure 6D). We also examined miR-34c expression after lncRNA-AF147447 overexpressed and down-regulated and found that miR-34c was significantly higher when lncRNA AF147447 overexpressed and vice versa (Figure 6D). These results suggested that lncRNA-AF147447 and miR-34c was positively correlated *in vitro*. We also examined the correlation between miR-34c and Af147447 *in vivo* and found that they were also positively correlated (Figure 6D). Besides MUC2, EGFR and CD44 were also predicted to be miR-34c targets. We over-expressed or inhibit lncRNA-AF147447 and found that EGFR or CD44 was decreased after lncRNA AF147447 over-expressed and increased after lncRNA AF147447 inhibited (Supplementary Figure S3C and S3D). Taken together, these results demonstrated that lncRNA could regulate MUC2 expression not only by directing binding, but also through post-transcriptional pathway, such as regulating miRNA expression.

### *H. pylori* infection decreased lncRNA AF147447 expression by recruiting transcriptional factor E2F1

**Figure 7:**
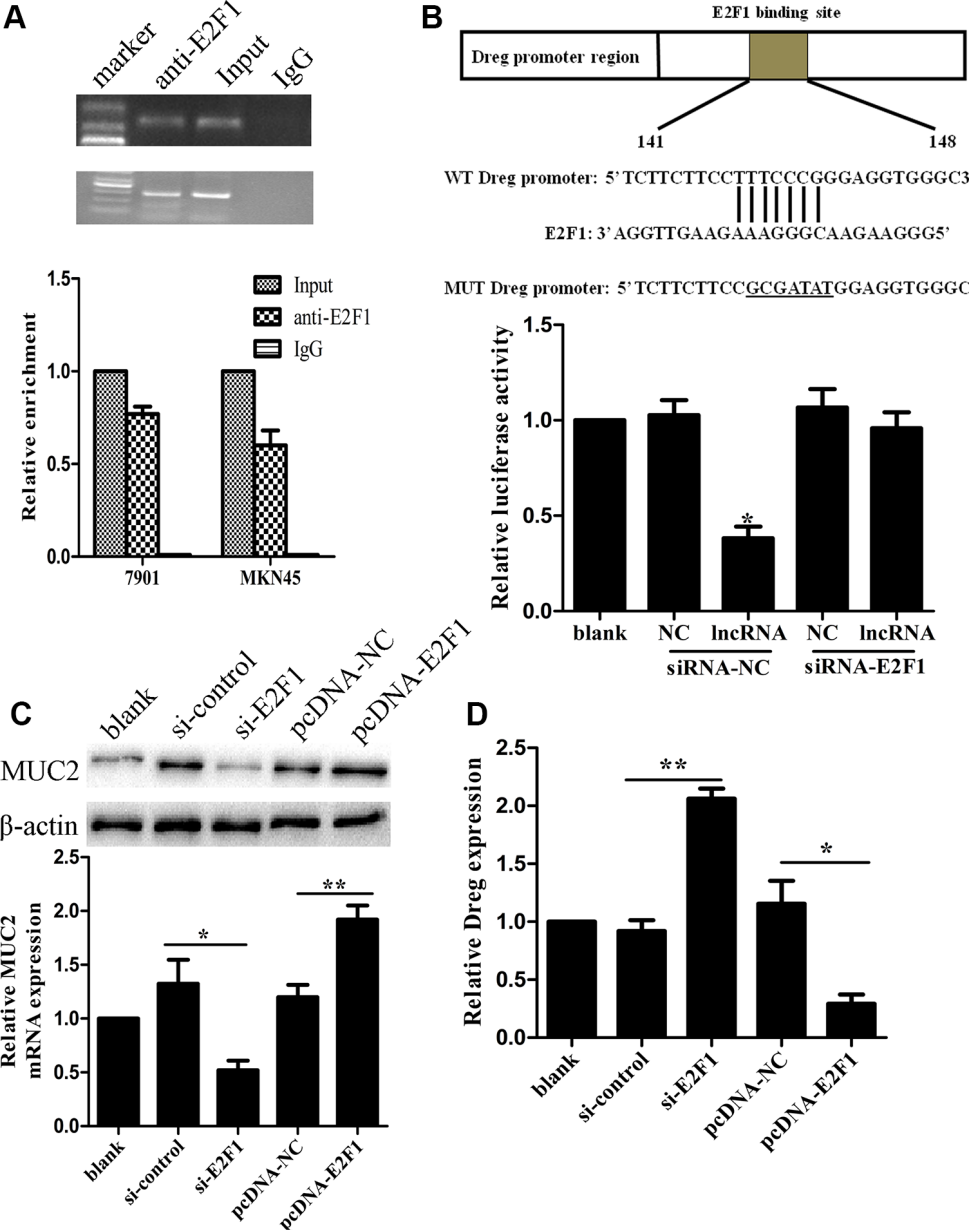
LncRNA AF147447 physically associates with E2F1 (**A**) RIP experiments were performed using the E2F1 antibody to immunoprecipitate (IP) and a primer to detect lncRNA-AF147447. RIP enrichment was determined as RNA associated with E2F1 IP relative to an input control. (**B**) Diagram of lncRNA-AF147447 promoter region constructs. Luciferase reporter assays in 7901 cells, with co-transfection of lncRNA-AF147447, with siRNA-E2F1 or siRNA-control. (**C**) LncRNA-AF147447 expression was detected by qRT-PCR after cells transfecting with si-E2F1 or pcDNA-E2F1. (**D**) MUC2 mRNA and protein were detected by qRT-PCR and western blot. (*p < 0.05; **p < 0.01).

A computational screen was conducted and predicted that transcriptional factor E2F1 was localized within AF147447 transcriptional element (Supplementary Figure S3). Recent studies have reported that transcription factors can bind to the promoter region of target lncRNAs and inhibit their expression. We wondered whether down-expression of AF147447 was mediated by this transcriptional factor. Firstly, to validate whether E2F1 could recruit to AF147447 promoter region and direct interaction of lncRNA AF147447 with E2F1, we performed RIP-qPCR and the results demonstrated that the lncRNA AF147447 RIP is significantly enriched for E2F1 compared to IgG (Figure 7A). The association between lncRNA AF147447 and E2F1 was further validated by luciferase reporter analysis. We constructed AF147447 wt and mut promoter and found that lncRNA AF147447 transfection could significantly reduce the luciferase activity, which could be reversed by si-E2F1 co-transfection (Figure 7B). Next, we investigated the functional association between E2F1 and lncRNA AF147447. After transfecting with si-E2F1, we found that lncRNA AF147447 was significantly over-expressed while transfecting with pcDNA-E2F1, lncRNA AF147447 was significantly reduced (Figure 7C), which suggested that E2F1 could change lncRNA AF147447 expression. We further examined whether E2F1 transfection could alter target gene MUC2 expression and found that MUC2 was significantly lower when si-E2F1 transfected and higher when pcDNA-E2F1 transfected (Figure 7D). We then validated the correlation between E2F1 expression *in vivo* with lncRNA AF147447 and MUC2. The results showed that E2F1 expression was negatively correlated with lncRNA AF147447 (Supplementary Figure S4A) and positively correlated with MUC2 (Supplementary Figure S4B).

## DISCUSSION

In this study, as shown in Supplementary Figure S4C, we reported that after *H. pylori* infection, lncRNA AF147447 expression level was decreased and its down-regulation could inhibit GC cell proliferation and invasion by inducing target oncogene, MUC2. Therefore, lncRNA AF147447 plays an important role in *H. pylori* infection-related GC. We also found that lncRNA AF147447 was markedly decreased in GC and was further decreased in *H. pylori* positive GC. By applying gain-of-function and loss-of-function methods, lncRNA AF147447 was identified to significantly inhibit cell proliferation and invasion. Additionally, it was shown that the expression of lncRNA AF147447 was regulated by E2F1 and oncogene MUC2 was a direct target of AF147447. It can also been found that AF147447 could post-transcriptionally affect MUC2 expression by binding with miR-34c.

GC is a worldwide malignant disease with a high incidence, high recurrence and poor prognosis, especially in Southeast Asia [12]. Significant proportion of the GC patients was *H. pylori* infected. Consequently, comprehensively study the mechanism and then develop new targeted treatments for *H. pylori* infection related GC was indeed necessary. Epigenetic modification belongs to one of the many characteristics of early process of tumorigenesis, changing the surrounding microenvironment to form a tumor [13]. Protein coding and non-protein coding genes (microRNA, lncRNA, circRNA) dysregulation has been identified to be able to diagnosing and predicting GC patients' prognosis [14]. However, whether *H. pylori* infection could promote GC through lncRNA regulation has not been previously elucidated. In the present study, we found that patients with the severe histology had much lower lncRNA AF147447 expression than normal controls. Previous reports showed that deregulated lncRNAs in GC could also be used as prognostic and diagnostic biomarkers [15]. Detecting the expression level of these lncRNAs, in combination with lncRNA AF147447 and even protein-coding genes or miRNAs, may be valuable to predict the prognosis of *H. pylori* related patients more accurately [16]. Furthermore, like some other lncRNAs, for example M86523 identified in our microarray analysis, are also suggested to be differentially expressed in positive and negative tissues, we will next determine whether deregulation of these lncRNAs also correlates with the histology of the patients and whether detecting these lncRNAs together is more precise in identifying the prognosis of GC patients [16].

We further examined the role of AF147447 *in vitro* and we found that AF147447 could inhibit tumor proliferation and invasion. We then explored that GC cells with over-expression or down-regulation of AF147447 might play a critical role in tumor growth *in vivo*. Furthermore, an RNA pull-down assay syggested that AF147447 was able to combine with transcription factor E2F1 and its expression level was mediated by E2F1. E2F1 was a transcription factor which over-expressed by *H. pylori* infection [17]. Transcription factor could combine with the promoter of the gene and suppress its expression [18]. We have also identified a target gene, MUC2, as affected by AF147447 down-regulation. The mucin core proteins (MUCs) are glycosylated proteins expressed in tissue-specific patterns in the gastrointestinal tract [19]. It has been found over-expressed in various cancers, such as colorectal cancer [20], ovarian cancer [21]. MUC2 over-expression is an adverse prognostic factor in cancers [22]. The regulatory role of MUC2 in tumor progression suggests that MUC2 could be considered as a new diagnostic marker for cancer diagnosis and a new drug target for the cancer therapy [23]. We also found that lncRNA AF147447 could regulate MUC2 expression by regulating miR-34c expression. From pearson correlation analysis, we found that miR-34c and lncRNA AF147447 was positively correlated in tissues. Besides, lncRNA AF147447 transfection could alter miR-34c expression and vice versa.

As some studies have shown that *H. pylori* infection could change the expression of certain kinds of lncRNA [24], our current study is one of the important supplements to the *H. pylori* related carcinogenesis, which provided a brand new lncRNA diagnostic biomarker and therapeutic target for GC. However, the roles of other differentially expressed lncRNAs identified from the microarray analysis need further validation and analysis.

## MATERIALS AND METHODS

### Patient samples

Seventy-five pairs of *H. pylori* positive and negative tissues were obtained from patients who underwent gastroscrope and 50 pairs of *H. pylori* positive and negative tumor tissues were obtained from patients who underwent gastrectomy in Jiangsu province Hospital. The basic characteristics of the enrolled patients were listed in Supplementary Table S1. For the use of materials for research purposes, written informed consent was obtained from each patient. The consent procedure and study protocol were approved by the Medical Institutional Ethical Committee of first affiliated hospital of Nanjing medical university.

### Microarray detection and bioinformatics analyses

Three pairs of gastric tissues from *H. pylori* positive and negative patients were randomly selected for microarray analysis. Total RNA was extracted, amplified, and transcribed into fluorescent cDNA. Labeled samples were hybridized to the Human LncRNA Array. Differentially expressed lncRNAs with statistical significance were identified through Volcano Plot filtering. The threshold to screen up- or down-regulated lncRNAs was fold change > 2 and *P* < 0.05.

### Cell culture

Human gastric cancer cell lines 7901 and MKN45 were obtained from American Type Culture Collection (ATCC). All cell lines were maintained in RPMI 1640 supplemented with 10% fetal calf serum at 37°C in a humidified atmosphere of 95% air and 5% CO_2_. Cell lines have identified by authentication.

### Cell proliferation assay and transwell invasion assay

Cell proliferation was assayed using CCK8 (Roche, Basel, Switzerland). Invasion was assessed using the *in vitro* assay, as described previously [22].

### *In vivo* assays for tumor growth

Cells (1 × 10^7^) that had been stably transfected with pLV-AF147447/pLV-NC or transient transfected with siRNA/control were suspended in 100 μL PBS and implanted subcutaneously into the bilateral armpit of BALB/c nude mice. The tumors were measured every three days after implantation, and the volume of each tumor was calculated by length × width^2^ × 0.5.

### Quantitative real-time PCR

Total RNA was extracted using Trizol reagent (TAKARA, Japan). First-strand cDNA was generated using the Reverse Transcription System Kit (TAKARA). Random primers were used for RT-PCR for lncRNAs since some lncRNAs do not have poly A tails. Quantitative real-time PCR was performed using a standard SYBR-Green PCR kit protocol on a StepOne System (Applied Biosystems, Foster, CA). GAPDH was used as an endogenous control. The primers were shown in Supplementary Table S3. qRT-PCR was performed using ABI Prism 7900HT (Applied Biosystems, Foster City, CA, USA) according to the direction of the reagents. The details are as described previously [22].

### Western blot analysis

Total cell lysate was prepared in 1×SDS buffer. Equivalent amounts of proteins were separated by SDS-PAGE and transferred onto NC membranes (Millipore, USA). After incubation with antibodies specific for either MUC2 (abcam, USA) or β-actin (Cell signaling Technology), blots were incubated with IRDye800 anti-rabbit secondary antibodies. The integrated density of the band was quantified by the ImageJ software (NIH, Bethesda, MD, USA).

### Immunohistochemical assay

Sections were deparaffinized rinsed in PBS twice and incubated with 10% normal goat serum for 30 min to block non-specific antibody binding. After washing, samples were incubated with primary anti-rabbit antibody MUC2 (Abcam, USA) and then incubated with secondary antibodies. Then, the sections were stained with DAB according to the manufacturer's protocols and mounted and photographed using a digitalized microscope camera (Nikon, Tokyo, Japan).

### Rna pull-down assay

LncRNA-AF147447 and its antisense RNA were *in vitro* transcribed from vector pLV-AF147447 and biotin-labeled with the Biotin RNA Labeling Mix (Roche Diagnostics, Indianapolis, IN) and T7/SP6 RNA polymerase, treated with RNase-free DNase I (Roche), and purified with an RNeasy Mini Kit (Qiagen, Valencia, CA). One milligram of protein from SGC-7901 cells stably transfected with pLV-AF147447 extracts was then mixed with 50 pmol of biotinylated RNA, incubated with streptavidin agarose beads (Invitrogen, Carlsbad, CA), and washed. The retrieved proteins were resolved by SDS-PAGE) and the specific bands were excised and analyzed by mass spectrometry and western blot.

### RNA immunoprecipitation

RIP experiments were performed using a Magna RIP RNA-Binding Protein Immunoprecipitation Kit (Millipore, Bedford, MA) according to the manufacturer's instructions. Antibody for RIP assays of E2F1 (Cell Signaling Technology, Beverly, MA) was diluted 1:1000. Coprecipitated RNAs were detected by RT-PCR. Gene specific primers used for detecting AF147447 are presented in Supplementary Table S3.

### Luciferase assay

AF147447 or MUC2 promoter regions were amplified by PCR and cloned downstream of the firefly luciferase gene in pGL3 vector (Promega, USA). The vector was named wild-type (wt) promoter regions. Site-directed mutagenesis of the E2F1 or AF147447 binding site in the promoters was generated by Invitrogen and named mutant (mut) promoter regions. For reporter assays, wt or mut vector and the control vector pRL-CMV were co-transfected. Luciferase activity was measured 36 hours after transfection using the Dual-Luciferase Reporter Assay System (Promega).

### Statistical analysis

Statistical analysis was performed using SPSS software (SPSS, Chicago, Illinois, USA). Comparisons between two groups were performed using Student's *t-test* or Mann–Whitney *U* test, as appropriate. All values were expressed as mean ± SD. Two-tailed *t* tests were applied to all data unless otherwise specified, with *P* < 0.05 considered statistically significant.

## SUPPLEMENTARY MATERIALS


