# Supplementary material for: Helicobacter pylori infection related long noncoding RNA (lncRNA) AF147447 inhibits gastric cancer proliferation and invasion by targeting MUC2 and up-regulating miR-34c
Source: Oncotarget. 2016 Nov 7;7(50):82770–82. doi: 10.18632/oncotarget.13165 (PMC5347731; doi:10.18632/oncotarget.13165)
Supplement: Supplementary file 1 [file oncotarget-07-82770-s001.pdf]

Helicobacter pylori infection related long noncoding RNA (lncRNA) AF147447 inhibits gastric cancer proliferation and invasion by targeting MUC2 and up-regulating miR-34c

Supplementary Materials

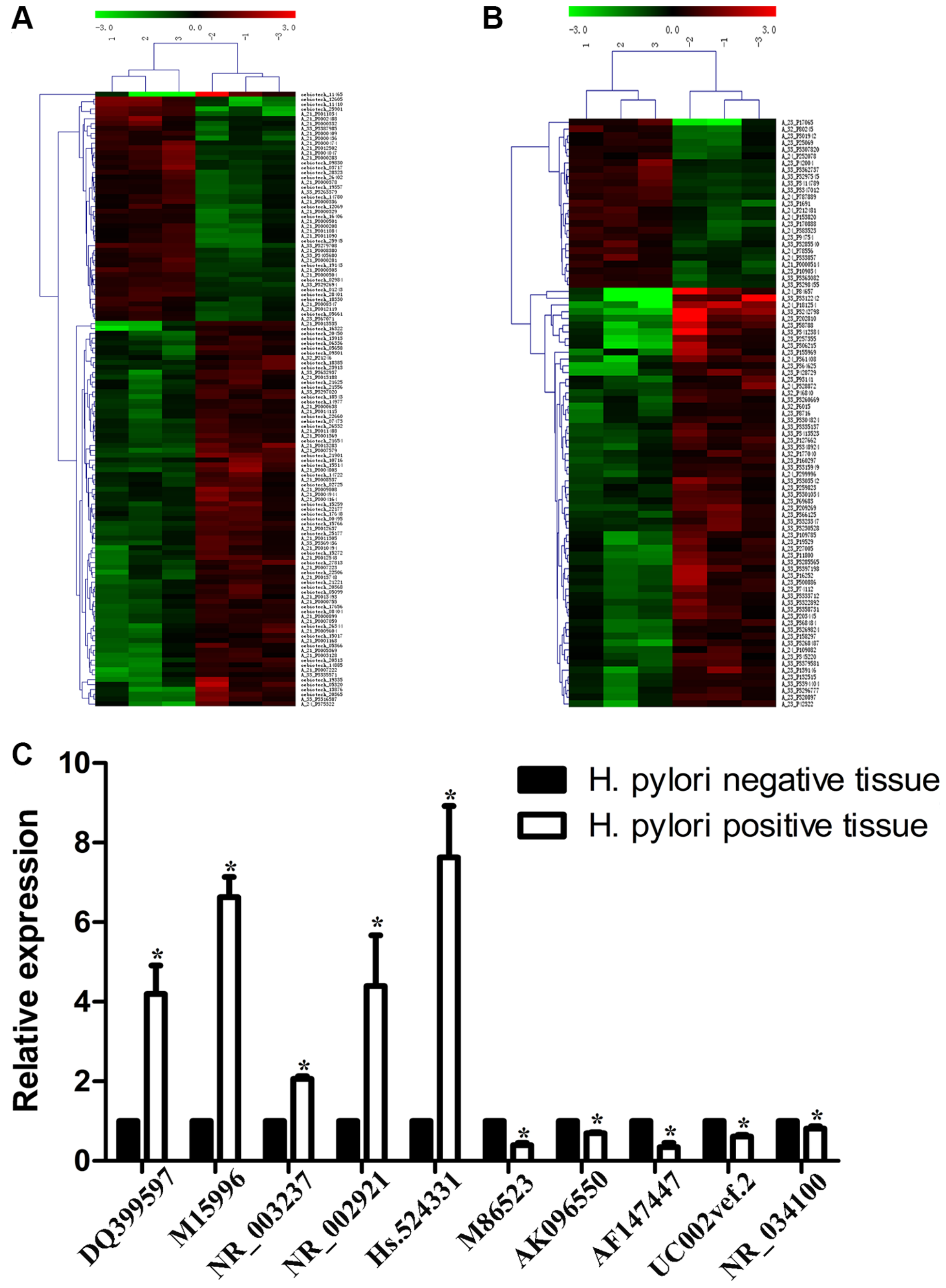

Supplementary Figure S1: Differential expression of lncRNAs in *H. pylori* positive and negative tissues. Hierarchical clustering analysis of 87 mRNAs (**A**) and 126 lncRNAs (**B**) that were differentially expressed between *H. pylori* positive and negative tissues. (greater than 2.0-fold; $P < 0.05$). Expression values are represented in shades of red and green, indicating expression above and below the median expression value across all samples (log scale 2, from –0.06 to +0.06), respectively. (**C**) We validated the differential expression of 10 lncRNAs in 3 paired *H. pylori* positive and control samples using RT-PCR. (*$p < 0.05$; **$p < 0.01$).

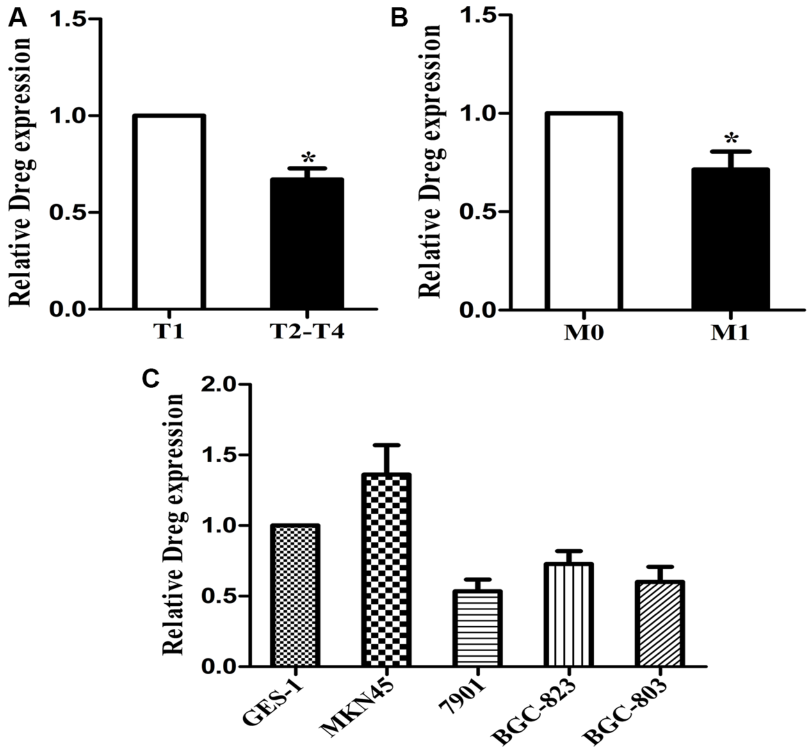

Supplementary Figure S2: LncRNA-AF147447 expression in GC cells and tissues. (**A**) LncRNA-AF147447 expression was validated by qRT-PCR in 5 different gastric epithelial cells. (**B**) LncRNA-AF147447 expression was examined in different GC stages in patients. (**C**) LncRNA-AF147447 expression was examined in different metastasis states.

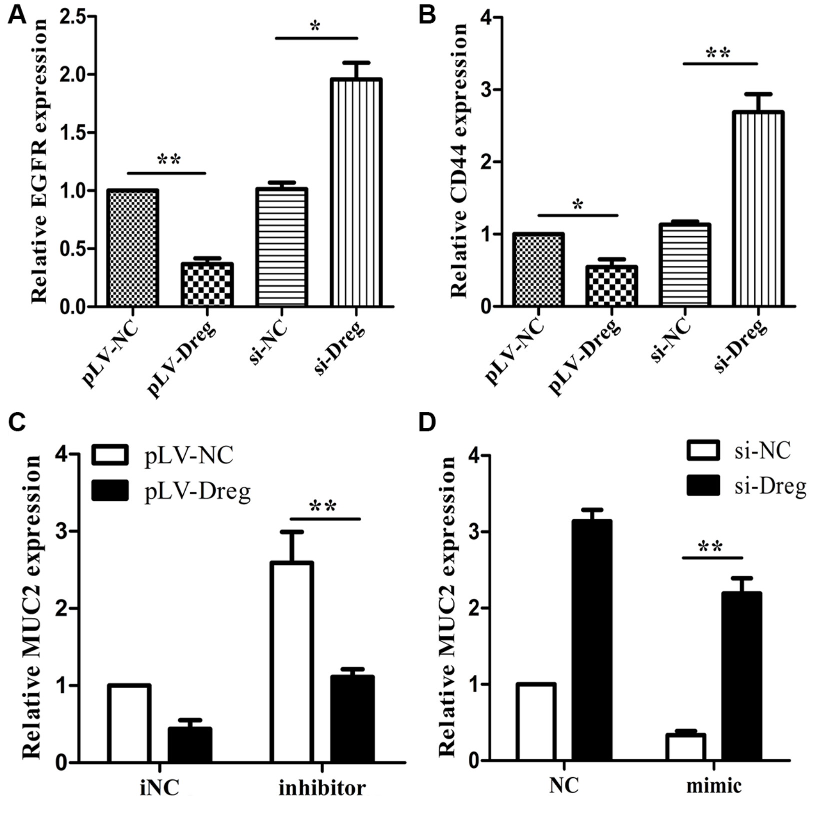

Supplementary Figure S3: Target gene expression after transfecting with AF147447 or miR-34c. (**A**) EGFR or CD44 mRNA expression after cells transfecting with pLV-AF147447 or si-AF147447. (**B**) MUC2 mRNA expression after cells transfecting with mimic or inhibitor with or without lncRNA AF147447. (*$p < 0.05$; **$p < 0.01$). (**C**) MUC2 mRNA expression was detected after co-tranfect miR-34c inhibitor/iNC and PLV-AF147447/NC. (**D**) MUC2 mRNA expression was detected after co-tranfect miR-34c mimic/NC and si-AF147447/iNC.

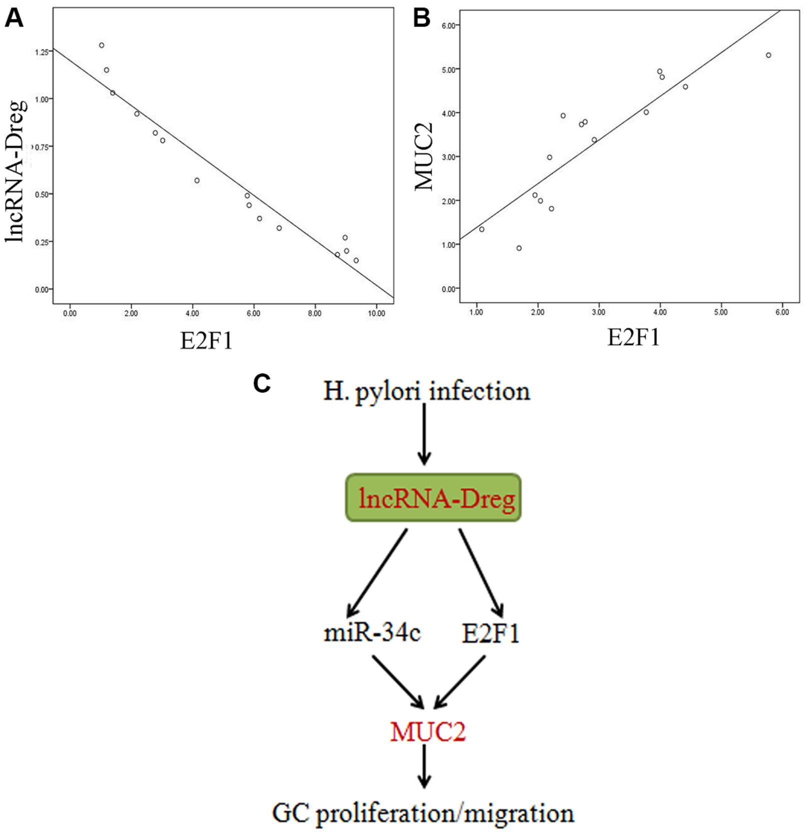

Supplementary Figure S4: Pearson correlation. (**A**) A statistically significant inverse correlation between E2F1 and lncRNA-Af147447 levels in clinical specimens. (**B**) A statistically significant positive correlation between E2F1 and MUC2 in clinical specimens. (**C**) The proposed mechanisms by which lncRNA AF147447 involved in *H. pylori*-induced GC.

Supplementary Table S1: Clinical characteristics of the enrolled control and tumor patients

Factor	Hp (+) control	Hp (−) control	*P* value
population	75	75	
Gender			
Male	41	39	0.435
Female	34	36	
Age(years)			
< 40	31	33	0.434
≥ 40	44	42	
Smoking History			
Yes	33	21	0.03*
No	42	54	
Drinking History			
Yes	31	35	0.311
No	44	40	
Histology			
SG	45	54	0.084§
AG	21	14	
IM	9	7	

§SG/(AG + IM).

Factor	Hp (+) tumor	Hp (−) tumor	*P* value
population	50	50	
Gender			
Male	39	36	0.322
Female	11	14	
Age(years)			
< 40	13	14	0.500
≥ 40	37	36	
Smoking History			
Yes	21	19	0.419
No	29	31	
Drinking History			
Yes	23	26	0.345
No	27	24	
Stage			
T_1	11	14	0.322
T_2–T_4	39	36	
Metastasis			
M_0	26	28	0.421
M_1	24	22	

Supplementary Table S2: Mass spectrometry analysis of the proteins pulled down by lncRNA-AF147447

No	Gene symbol	Gene description	Nondegenerative sequence	Relative abundance
1	MUC2	mucin 2, oligomeric mucus/gel-forming [homo]	Yes	38.1%
2	ILF2	Interleukin enhancer-binding factor 2 [homo]	Yes	33.1%
3	TRAP1	TNF receptor-associated protein 1 [homo]	Yes	7.1%
4	RPS3	40S ribosomal protein S3	No	7.2%
5	CDK2	cyclin-dependent kinase 2	Yes	5.9%
6	unknown	Unnamed protein product	Yes	3.1%
7	HDAC1	histone deacetylase 1	Yes	3.1%
8	ABCB1	ATP-binding cassette, sub-family B (MDR/TAP), member 1	No	2.4%

Supplementary Table S3: Sequences of primers and siRNA used in this study

Name		Sequences
**qPCR primers**		
DQ399597	Sense	5′-CCTGTATGACGATGGAGCCT-3′
	Anti-sense	5′-TGACACATTTGCGATGGGTAT-3′
M15996	Sense	5′-GACCGACAAGGGATGGAC-3′
	Anti-sense	5′-ATTCTGAGTAACAGGAGCACAC-3′
TCONS-L2-00005493	Sense	5′-CAACCTGGCAGCATCTACAAATA-3′
	Anti-sense	5′-ATGGTGGCTCACAACCCTCT-3′
Hs.524331	Sense	5′-AATCTTCCTTCCCTTGAGCC-3′
	Anti-sense	5′-CAGGTGAATCGTTATTTATTATCCC-3′
ENST00000538098	Sense	5′-GATACCTGACCCACTGTTCCC-3′
	Anti-sense	5′-ATGCCTTGGCTCATTCTTTG-3′
M86523	Sense	5′-TGGGAAACTGCCATAGAAGAT-3′
	Anti-sense	5′-GTTGGGAGGGAAAGGGTG-3′
TCONS-L2-00028472	Sense	5′-CGCACATCTATTGCTCGTCC-3′
	Anti-sense	5′-AAGCCCACAGTTCAGCCATC-3′
AF147447	Sense	5′-TCCTCTAATGCGTCTTGTCTCC-3′
	Anti-sense	5′-CCCATACCAAACTCTAACCACC-3′
AK096550	Sense	5′-TACAACTCGGAGCATTCAAGC-3′
	Anti-sense	5′-TGTCAAGCACAGCAGCAAGC-3′
uc002vef.2	Sense	5′-CAGAATCAAATGCCACCACAGA-3′
	Anti-sense	5′-GCTAAACCAGACAAGGCTCCC-3′
MUC2	Sense	5′-GAGGTGGAGCGGGACAA-3′
	Anti-sense	5′-GCAGGGTGCTTTCGGC-3′
GAPDH	Sense	5′-TGTGTTGGCGTACAGGTCTTTG-3′
	Anti-sense	5′-GGGAAATCGTGCGTGACATTAAG-3′
CD44	Sense	5′-CTGCCGCTTTGCAGGTGTA-3′
	Anti-sense	5′-CATTGTGGGCAAGGTGCTATT-3′
EGFR	Sense	5′-AGGCACGAGTAACAAGCTCAC-3′
	Anti-sense	5′-ATGAGGACATAACCAGCCACC-3′
E2F1	Sense	5′-GCTAACGAACAAAGCCAGA-3′
	Anti-sense	5′-CCCTATTCTCATGCAAGGA-3′
**siRNA sequences**		
Dreg siRNA1	Sense	5′-CCUCCCAGGUUCAAGUAAUTT-3′
	Anti-sense	5′-AUUACUUGAACCUGGGAGGTT-3′
Dreg siRNA2	Sense	5′-CAGGCUUGGUAACUAACAUTT-3′
	Anti-sense	5′-AUGUUAGUUACCAAGCCUGTT-3′
Dreg siRNA3	Sense	5′-GCUUCUUCUAUUCUCUCUUTT-3′
	Anti-sense	5′-AAGAGAGAAUAGAAGAAGCTT-3′
siRNA NC	Sense	5′-GUGGGCAACAUUCUUCGAATT-3′
	Anti-sense	5′-UUCGAAGAAUGUUGCCCACTT-3′

The sequence of pLV-Dreg:
TTTTTTTTTTAGAATTTATTTTTATTCTTTCAGACCTCTCAGGGATGAACGGACATAGGCTTCTAACACTTAGGTGTGGGC
AACATTCTTCGAAGCACCTCCCTCAAAGTGGAAAAGGCCTGGGGGCTCAGACGAGAGAAGAGAGAAGGCAGGGAGA
AGGTGGAGGTGAGGAAGGGAGGAGGGGGCCCAGGGCTCAGTGGGGTGTTGGGGAGGGTGGGATTCCACCGGGGTT
TGCCCATCCACAGCTCAGTGGGGGGATCTATGGAGTGTGTCTAGCAAGAGAGGACCCATGGCAGGCTTGGTAACTAAC
ATCATGCAGTAGCTTCTTCTATTCTCTCTTATTTTTTTGGAAACGGAGTCTCGCTCTTGTTGCCCAGGCTGGAGTACAGT
GGCACAATCTCAGCTCACTGCAACCTCTGCCTCCCAGGTTCAAGTAATTC.
